# A new prognostic score for disease progression and mortality in patients with newly diagnosed primary CNS lymphoma

**DOI:** 10.1002/cam4.2872

**Published:** 2020-02-03

**Authors:** Chia‐Jen Liu, Shinn‐Yn Lin, Ching‐Fen Yang, Chiu‐Mei Yeh, Ai‐Seon Kuan, Hao‐Yuan Wang, Chun‐Kuang Tsai, Jyh‐Pyng Gau, Liang‐Tsai Hsiao, Po‐Min Chen, Yao‐Chung Liu, Ying‐Chung Hong, Po‐Shen Ko, Jin‐Hwang Liu, Chia‐Hsin Lin

**Affiliations:** ^1^ Division of Hematology and Oncology Department of Medicine Taipei Veterans General Hospital Taipei Taiwan; ^2^ Institute of Public Health National Yang‐Ming University Taipei Taiwan; ^3^ Department of Radiation Oncology Linkou Chang Gung Memorial Hospital Medical Center Taoyuan City Taiwan; ^4^ Department of Medical Imaging and Radiological Sciences College of Medicine Chang Gung University Taoyuan Taiwan; ^5^ Department of Pathology and Laboratory Medicine Taipei Veterans General Hospital Taipei Taiwan; ^6^ Cancer Epidemiology Unit Nuffield Department of Population Health University of Oxford Oxford UK; ^7^ School of Medicine National Yang‐Ming University Taipei Taiwan; ^8^ Division of Hematology and Oncology Kaohsiung Veterans General Hospital Kaohsiung Taiwan; ^9^ Institute of Biopharmaceutical Sciences National Yang‐Ming University Taipei Taiwan; ^10^ Chong Hin Loon Memorial Cancer and Biotherapy Research Center National Yang‐Ming University Taipei Taiwan

**Keywords:** epidemiology, external validation, primary CNS lymphoma, prognostic model

## Abstract

**Background:**

Although various prognostic models for primary central nervous system lymphoma (PCNSL) have been developed, there is no consensus regarding the optimal prognostic index. We aimed to evaluate potential prognostic factors and construct a novel predictive model for PCNSL patients.

**Methods:**

We enrolled newly diagnosed PCNSL patients between 2003 and 2015. The primary endpoint was progression‐free survival (PFS), and the secondary endpoint was overall survival (OS). The prognostic factors identified using multivariate Cox proportional hazards models were used to develop a predictive model. We subsequently validated the prognostic model in an independent cohort. We also evaluated the validity of the existing scores: the International Extranodal Lymphoma Study Group (IELSG), the Nottingham/Barcelona (NB), and the Memorial Sloan‐Kettering Cancer Center models (MSKCC).

**Results:**

We identified 101 patients with newly diagnosed PCNSL at our center. Multivariate analysis showed that age ≥80, deep brain lesions, and ECOG ≥2 were independent risk factors of PFS. Assigning one point for each factor, we constructed a novel prognostic model, the Taipei Score, with four distinct risk groups (0‐3 points). The performances of the Taipei Score in discriminating both PFS and OS in the training cohort were significant, and the score was validated in the external validation cohort. The IELSG, NB and MSKCC models had insufficient discriminative ability for either PFS or OS in both cohorts.

**Conclusion:**

The Taipei Score is a simple model that discriminates PFS and OS for PCNSL patients. The score may offer disease risk stratification and facilitate clinical decision‐making.

## Graphical abstract

Age, performance status, and deep brain lesions were associated with primary central nervous system lymphoma (PCNSL) survival. The Taipei Score could offer disease risk stratification for PCNSL.

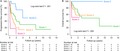

## 1 INTRODUCTION

Primary central nervous system lymphoma (PCNSL) is a relatively rare malignant neoplasm, representing around 4% of all primary CNS tumors.^1^, ^2^ Although evolving therapeutic strategies have significantly improved overall survival (OS) of some patients, many patients still succumb to this disease due to its high propensity to recur.^3^, ^4^ The wide range of survival for PCNSL patients (from a few months to several years) underscores the need to develop a reliable disease prognostic model that may predict disease outcomes, and facilitate decision‐making for further treatments. In addition, given the low incidence of PCNSL, large randomized phase III trials have been relatively lacking regarding optimal standard treatment, of which the consensus is therefore based mainly on the comparative analysis of retrospective studies and phase II trials.^4^, ^5^, ^6^ It is thus of great importance to develop a reliable prognostic model to help compare multiple studies and even assist in the design of a proper stratification guideline for future phase III clinical trials.

Many studies have examined prognostic factors for PCNSL, with age and performance status (PS) being the only two factors that were consistently reported to be associated with disease survival.^7^, ^8^, ^9^, ^10^, ^11^ To date, three disease prognostic models developed in Western populations can estimate the survival of PCNSL patients. The first model was developed by the International Extranodal Lymphoma Study Group (IELSG), encompassing five variables, namely age, Eastern Cooperative Oncology Group (ECOG) performance status, serum lactate dehydrogenase (LDH), level of cerebrospinal (CSF) protein, and deep brain involvement (ie, periventricular regions, basal ganglia, brainstem, and/or cerebellum).^9^ Although the IELSG model was established based on a relatively large sample of patients (n = 378) from multiple centers, two‐thirds of the samples lacked data on LDH level or CSF protein. Information regarding LDH level or CSF protein was not always obtainable in the clinical practice, making the application and validation of IELSG difficult in many previous studies.^11^, ^12^, ^13^, ^14^ The second model is a three‐factor scoring system developed by researchers in Nottingham and Barcelona, consisting of age, PS, presence of multifocal lesions or meningeal disease.^10^ The Nottingham/Barcelona (NB) score was developed in a relatively small patient population (n = 77) that received old chemotherapy regimens, so its application to today's PCNSL populations is limited. Additionally, the NB score is unable to distinguish survival outcomes for two of its risk‐stratified groups (score 1 group vs. score 2 group). The third prognostic score was developed by researchers at Memorial Sloan‐Kettering Cancer Center (MSKCC), and it includes two variables only, namely patient age and Karnofsky performance status (KPS).^11^ Although the external validity of the MSKCC model was shown in the original publication, several recent studies had failed to associate the score with PCNSL survival,^12^, ^15^ which raises doubts about the reliability of this two‐parameter model.

Moreover, treatments for PCNSL and survival of patients have progressed much over time after the development of the three prognostic models. The prognostic value of these existing models may also change over time due to improved therapy and supportive care. Therefore, we constructed a new prognostic score that caters to PCNSL patients in more recent periods using data from two medical centers in Taiwan. The data of PCNSL patients diagnosed at Taipei Veterans General Hospital was used to develop a new prognostic score—the Taipei Score. We validated the Taipei Score using data from Linkou Chang Gung Memorial Hospital. We also used the two cohorts to validate the IELSG, NB and MSKCC models.

## 2 METHODS

### 2.1 Data sources

All patients who were newly diagnosed with PCNSL and followed up at Taipei Veterans General Hospital, the largest public medical center in Taiwan, from 1 January 2003 to 31 December 2015, were recruited into our study to develop the scoring system. The inclusion criteria were the following: (a) histopathologically verified non‐Hodgkin's lymphoma, and (b) disease involving exclusively the brain, cranial nerves, leptomeninges, or eyes. We excluded patients who had human immunodeficiency virus seropositivity, other immunodeficiency diseases or evidence of systemic non‐Hodgkin's lymphoma from computed tomography (CT), magnetic resonance imaging (MRI), or positron emission tomography–computed tomography (PET/CT) images of the chest, abdomen, pelvis, bone marrow aspiration, or biopsy. The cohort was followed up until the end of February 2017.

The institutional review boards of both Taipei Veterans General Hospital and Linkou Chang Gung Memorial Hospital approved this study (no. 2016‐05‐003BC and 201900184B0, respectively).

### 2.2 Data collection

Information including age, sex, date of PCNSL diagnosis, date of death, comorbidities, ECOG, tumor location, LDH levels, CSF protein, hemoglobin levels, C‐reactive protein, and bilirubin levels were collected retrospectively by reviewing each patient's inpatient and outpatient medical records. ECOG of 2 was used as cutoff according to prior studies of PCNSL.^9^, ^10^ Similarly, age of 80 years was chosen as cutoff according to previous research showing 80 years as a potential risk factor for mortality in patients with systemic diffuse large B‐cell lymphoma^16^, ^17^ as well as PCNSL.^18^ Cutoff values of hemoglobin, C‐reactive protein, and bilirubin levels were 11.85 g/dL, 2.94 mg/dL, and 0.52 mg/dL, respectively, which were correlated with clinical outcomes in one previous study of diffuse large B‐cell lymphoma.^19^ Information on cause of death and disease status was identified at the end of follow‐up. The primary endpoint was progression‐free survival (PFS), which was calculated from the date of pathologic diagnosis of PCNSL to the date of progression, relapse, or death from any cause.^20^ The secondary endpoint was OS, which was defined as the time from pathologic diagnosis of PCNSL to any‐cause death.

### 2.3 IELSG, NB and MSKCC models

The IELSG model is based on five variables, including age >60, ECOG >1, increased serum LDH, elevated CSF protein concentration, and deep brain involvement. Each variable is assigned a value of either 0 if favorable, or 1 if unfavorable, of which the sum yields the final score (0‐1 score group; 2‐3 score group; 4‐5 score group). The NB model is composed of three risk factors, including age ≥60, ECOG >1, and presence of multifocal lesions or meningeal disease. Each risk factor is assigned one point, giving rise to four risk‐score groups (0, 1, 2, and 3). The MSKCC model consists of two prognostic variables only, namely age and KPS, which defines three prognostic classes: class 1 (age <50), class 2 (age ≥50 and KPS ≥70), and class 3 (age ≥50 and KPS <70).

### 2.4 Validation cohort

A different cohort of consecutive patients with PCNSL treated at Linkou Chang Gung Memorial Hospital, the largest private hospital in Taiwan, from 1 January to 31 December 2017, made up the independent external validating cohort. The inclusion and exclusion criteria of this cohort were the same as that of our training cohort. The prognostic score that we developed and the aforementioned three models were tested in the validation cohort.

### 2.5 Statistical analysis

Baseline characteristics of study participants were summarized as frequencies and percentages for categorical data, and medians and range for continuous data. Continuous data and categorical data between participants in the training cohort and validation cohort were compared using the Mann—Whitney *U* test and chi‐square test, respectively. A univariate Cox proportional hazard model was used to calculate hazard ratios (HRs) and 95% confidence intervals (CIs) for disease progression and death associated with patient characteristics in our cohort. Factors with *P* values < .1 in the univariate model were selected for inclusion in the multivariate analysis. Then we defined the significant risk factors when it significantly correlated with PFS or OS in the multivariate analysis. We subsequently built a risk score incorporating risk factors with *P* < .05 and assigned one point for each factor, yielding a final score. We then validated the new score in the validation cohort. We estimated the PFS and OS using the Kaplan—Meier method, and compared the PFS and OS by the risk score using a log‐rank test. The discriminative ability of the new model was also evaluated with Harrell's C‐statistics. C‐statistic equivalent to 0.5 indicates no predictive discrimination and a value of 1.0 indicates perfect discriminative ability.^21^ The IELSG, NB and MSKCC models were also tested using the same methods.

All statistical tests were two‐sided and a *P* value of < .05 was defined as statistically significant. Analyses were performed with the use of SAS software, version 9.4 (SAS Institute Inc), and STATA statistical software, version 15.1 (StataCorp).

## 3 RESULTS

### 3.1 Baseline characteristics of the PCNSL population in the training cohort

A total of 113 patients with CNS lymphoma diagnosed at Taipei Veterans General Hospital were identified. We excluded patients who were diagnosed with secondary CNS lymphoma (n = 7) or acquired immune deficiency syndrome (n = 5). Finally, 101 PCNSL patients were enrolled in the study for the training cohort. The pathological diagnosis of all patients in the training cohort was diffuse large B‐cell lymphoma. Baseline demographic characteristics of the PCNSL population are summarized in Table 1 and Table S1. Of the 101 patients in the training cohort, 58.4% were male, and the median age was 64 (range 22‐88 years). Fifty‐four patients (53.5%) had an ECOG PS of more than 1. The most common sites were the frontal lobe (38.6%) and basal ganglia (37.6%). The details of treatment are shown in Figure S1. Of the 101 PCNSL patients in the training cohort, 80 patients (79.2%) received chemotherapy as frontline therapy, and methotrexate (MTX) was the most commonly used drug (n = 76) in the first‐line treatment, followed by rituximab (n = 49), high‐dose cytarabine (n = 25), and vincristine (n = 16). Radiotherapy was administered in 55 patients (54.5%). Radiotherapy was delivered to the whole brain with a median dose (range) of 32 (6‐54) Gy, with or without a tumor‐bed boost with 15 (8‐36) Gy.

**Table 1:** Baseline characteristics of PCNSL patients

Characteristics	Training cohort n = 101	Validation cohort n = 81	*P* value
	n (%)	n (%)	
Median age, y (range)	64 (22‐88)	68 (17‐82)	.062
≥80	14 (13.9)	5 (6.2)	.092
Male sex	59 (58.4)	40 (49.4)	.224
Site			
Frontal lobe	39 (38.6)	45 (55.6)	.023
Parietal lobe	26 (25.7)	20 (24.7)	.871
Temporal lobe	34 (33.7)	22 (27.2)	.345
Occipital lobe	18 (17.8)	9 (11.1)	.206
Basal ganglia	38 (37.6)	21 (25.9)	.094
Brain stem	9 (8.9)	12 (14.8)	.215
Cerebellum	13 (12.9)	5 (6.2)	.133
Multifocal lesions	48 (47.5)	49 (60.5)	.081
Deep brain lesions	68 (67.3)	48 (59.3)	.261
ECOG			
0‐1	47 (46.5)	23 (28.4)	.012
≥2	54 (53.5)	58 (71.6)	
KPS			
<70	42 (41.6)	45 (55.6)	<.001
≥70	59 (58.4)	36 (44.4)	
Lactic dehydrogenase ≥250 U/L	43/92 (46.7)	11/57 (19.3)	.001
High CSF protein (>45 mg/dL)	42/57 (73.7)	27/41 (65.9)	.402

Abbreviations: CSF, cerebrospinal fluid; ECOG, Eastern Cooperative Oncology Group performance score; KPS, Karnofsky performance status; PCNSL, Primary central nervous system lymphoma.

### 3.2 Disease status and causes of mortality

At the time of last follow‐up, 69 patients (68.3%) were alive. Thirty‐two patients (31.7%) were deceased. Mortality was related to PCNSL with disease progression in 15 (46.9%), mass effect in 2 (6.3%), and tumor bleeding in 1 (3.1%) of deceased patients. Infections were also direct causes of mortality in 28.1% of deceased PCNSL patients (n = 9).Among patients who died of infection, 11.1% of patients died as a result of treatment‐related infection (n = 1). The causes of mortality are shown in Figure S2.

### 3.3 Risk factors for disease progression and mortality

In our multivariate analysis, we found that old age (≥80), presence of deep brain lesions, and poor performance status (ECOG ≥2) were significant risk factors for PFS in patients with PCNSL, with adjusted hazard ratios of 2.46 (95% CI 1.22‐4.99, *P* = .012), 2.57 (95% CI 1.39‐4.72, *P* = .003), and 1.87 (95% CI 1.09‐3.20, *P* = .024), respectively (Table 2). While age ≥80 (adjusted HR 2.94, 95% CI 1.15‐7.49, *P* = .024) and deep brain lesions (adjusted HR 2.53, 95% CI 1.07‐5.98, *P* = .035) were also significant risk factors for OS, worse ECOG was not (adjusted HR 1.61, 95% CI 0.76‐3.42, *P* = .212).

**Table 2:** Univariate and multivariate analysis of risk factors associated with PFS and OS in PCNSL patients (training cohort)

Predictive variables	PFS				OS			
	Univariate analysis		Multivariate analysis^a^		Univariate analysis		Multivariate analysis^a^	
	HR (95% CI)	*P* value	HR (95% CI)	*P* value	HR (95% CI)	*P* value	HR (95% CI)	*P* value
Age ≥80	2.35 (1.18‐4.67)	.015	2.46 (1.22‐4.99)	.012	2.72 (1.10‐6.71)	.030	2.94 (1.15‐7.49)	.024
Male sex	0.85 (0.51‐1.42)	.545			0.75 (0.38‐1.51)	.422		
Site								
Frontal lobe	0.63 (0.37‐1.08)	.090			0.91 (0.44‐1.85)	.788		
Parietal lobe	0.82 (0.46‐1.47)	.507			1.29 (0.61‐2.72)	.509		
Temporal lobe	1.06 (0.61‐1.82)	.848			1.30 (0.62‐2.72)	.484		
Occipital lobe	1.19 (0.61‐2.30)	.615			1.37 (0.56‐3.34)	.493		
Basal ganglia	2.52 (1.50‐4.23)	.001			4.16 (2.00‐8.64)	<.001		
Brain stem	1.58 (0.68‐3.69)	.289			0.84 (0.20‐3.51)	.807		
Cerebellum	0.98 (0.46‐2.06)	.955			1.00 (0.35‐2.86)	1.000		
Number of lesions ≥5	1.87 (0.85‐4.13)	.121			1.56 (0.54‐4.45)	.409		
Deep brain lesions	2.45 (1.34‐4.46)	.004	2.57 (1.39‐4.72)	.003	2.32 (1.00‐5.40)	.051	2.53 (1.07‐5.98)	.035
Midline shift	1.52 (0.90‐2.56)	.114			1.70 (0.83‐3.49)	.145		
Initial surgical treatment								
Stereotactic biopsy	Reference				Reference			
Open biopsy	0.51 (0.12‐2.11)	.352			0.42 (0.06‐3.09)	.391		
Partial resection	0.79 (0.45‐1.38)	.407			0.57 (0.25‐1.29)	.178		
CSF involvement	1.77 (0.53‐5.92)	.352			1.93 (0.24‐15.49)	.537		
Intraocular involvement	0.70 (0.28‐1.75)	.450			0.73 (0.19‐2.84)	.646		
ECOG ≥2	2.15 (1.26‐3.65)	.005	1.87 (1.09‐3.20)	.024	1.91 (0.92‐3.97)	.083	1.61 (0.76‐3.42)	.212
Lactate dehydrogenase ≥250 U/L	1.20 (0.71‐2.03)	.499			1.46 (0.70‐3.04)	.308		
High CSF protein (>45 mg/dL)	1.29 (0.61‐2.71)	.506			1.45 (0.46‐4.57)	.526		
Hemoglobin >11.85 g/dL	1.07 (0.65‐1.76)	.791			1.04 (0.59‐1.83)	.885		
CRP >2.94 mg/dL	0.95 (0.54‐1.68)	.862			1.22 (0.67‐2.23)	.512		
Bilirubin >0.52 mg/dL	0.97 (0.57‐1.66)	.908			0.75 (0.41‐1.37)	.348		

Abbreviations: CI, confidence interval; CRP, C‐reactive protein; CSF, cerebrospinal fluid; ECOG, Eastern Cooperative Oncology Group performance score; HR, hazard ratio; OS, overall survival; PFS, progression‐free survival.

a All factors with *P* < .1 in the univariate analysis were included in the Cox multivariate analysis. Then we defined the significant risk factors when it significantly correlated with PFS or OS in the multivariate analysis.

### 3.4 A new score for disease progression and mortality

**Figure 1:**
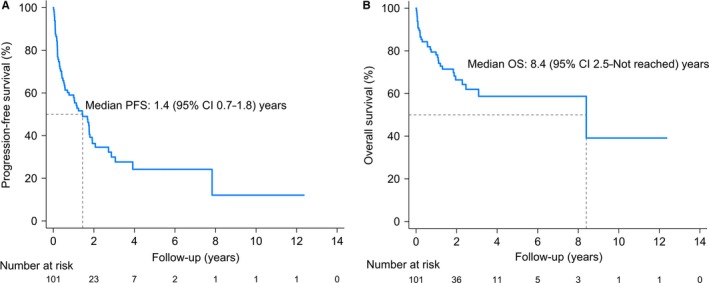
Kaplan—Meier estimates of (A) progression‐free survival and (B) overall survival among patients with primary central nervous system lymphoma (PCNSL; training cohort)

**Figure 2:**
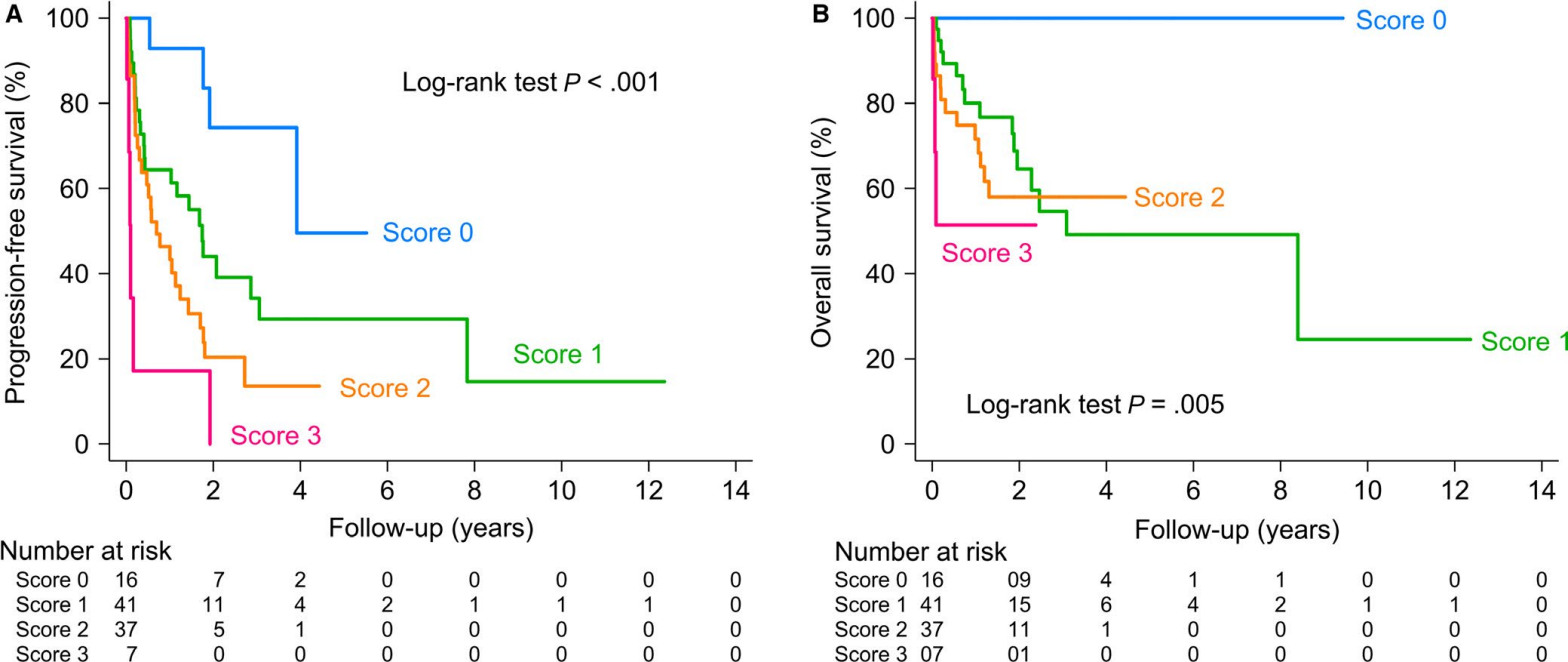
Kaplan—Meier estimates of (A) progression‐free survival and (B) overall survival in primary central nervous system lymphoma (PCNSL) patients with the Taipei Score (training cohort)

We developed a new score (the Taipei Score) for estimating PFS and OS among PCNSL patients, with a point given for each of the three significant factors (age ≥80, deep brain lesions and ECOG ≥2) found in our cohort. All 101 patients were found to be evaluable for the model. The prognostic score was significantly associated with the median PFS for the “0,” “1,” “2,” and “3” score groups, which were 3.9 years (95% CI, 1.8—not reached years), 1.7 years (95% CI, 0.4‐3.1 years), 0.7 years (95% CI, 0.3‐1.2 years), and 0.1 years (95% CI, 0.0—not reached years), respectively (Table 3). Likewise, it appears that higher scores were associated with worse OS (Table 3). Figure 1 shows Kaplan—Meier estimates for PFS and OS among patients with PCNSL in the cohort. The median PFS and OS were 1.4 years (95% CI 0.7‐1.8) and 8.4 years (95% CI 2.5 to not reached), respectively. In our cohort, PCNSL patients with higher scores had significantly shorter PFS (log‐rank test *P* < .001) and OS (log‐rank test *P* = .005) (Figure 2). Additionally, C‐statistic of the Taipei Score on the training cohort was 0.67 (95% CI 0.61‐0.74) for PFS and 0.67 (95% CI 0.59‐0.76) for OS (Table S2).

**Table 3:** Incidence of mortality or disease progression in PCNSL patients with risk scoring (training cohort)

	PFS					OS				
	Event no.	Per 100 PY	Median PFS (95% CI), y	HR (95% CI)	*P* value	Event no.	Per 100 PY	Median OS (95% CI), y	HR (95% CI)	*P* value
Taipei Score										
0	4	1.0	3.9 (1.8–a)	Reference		0	0.0	a	Reference	
1	23	2.8	1.7 (0.4‐3.1)	3.06 (1.05‐8.90)	.040	15	1.4	3.1 (1.9–a)	1.35 × 10^7^ (3.51–a)	.649
2	28	6.3	0.7 (0.3‐1.2)	5.34 (1.86‐15.33)	.002	14	2.2	a	1.64 × 10^7^ (4.23–a)	.040
3	6	20.9	0.1 (0.0–a)	15.37 (4.28‐55.25)	<.001	3	8.1	a	4.38 × 10^7^ (8.57–a)	<.001
IELSG prognostic score										
0‐1	3	1.6	1.9 (0.4–a)	Reference		1	0.4	a	Reference	
2‐3	17	2.6	1.7 (0.5–a)	1.57 (0.46‐5.39)	.475	9	1.0	7.8 (1.2–a)	2.06 (0.26‐16.50)	.496
4‐5	12	5.6	1.0 (0.3‐2.7)	2.76 (0.77‐9.84)	.117	6	1.7	1.7 (0.7–a)	3.04 (0.37‐25.34)	.304
NB prediction score										
0	3	1.8	a	Reference		2	1.1	a	Reference	
1	19	2.7	1.9 (1.2‐2.9)	1.39 (0.41‐4.70)	.600	10	1.0	7.8 (2.1–a)	1.07 (0.23‐4.90)	.931
2	27	4.1	0.8 (0.3‐1.8)	2.20 (0.67‐7.27)	.195	16	1.8	3.1 (0.7–a)	1.79 (0.41‐7.84)	.437
3	12	6.9	0.4 (0.2‐1.8)	2.96 (0.83‐10.51)	.093	4	1.5	a	1.26 (0.23‐6.87)	.793
MSKCC prognostic model										
Class 1	8	3.9	1.4 (0.3–a)	Reference		4	1.2	a	Reference	
Class 2	24	2.2	2.9 (1.0‐7.8)	0.65 (0.29‐1.47)	.306	14	1.0	7.8 (3.1–a)	0.82 (0.27‐2.51)	.723
Class 3	29	7.4	0.5 (0.2‐1.4)	2.20 (0.67‐7.27)	.195	14	2.4	2.1 (0.7–a)	1.40 (0.46‐4.25)	.557

Note

We specified risk strata by assigning one point for each of the three factors (age ≥80, deep brain lesions and ECOG ≥2) in the Taipei Score.

Abbreviations: CI, confidence interval; HR, hazard ratio; IELSG, International Extranodal Lymphoma Study Group; MSKCC, Memorial Sloan Kettering Cancer Center; NB, Nottingham‐Barcelona; OS, overall survival; PFS, progression‐free survival; PY, person‐years.

a Not reached.

### 3.5 Validation of the new score

Data from Linkou Chang Gung Memorial Hospital were used for external validation (n = 81). Of the 81 patients, 40 patients (49.4%) were men, and the median age was 68 (range 17‐82) years. Fifty‐eight patients (71.6%) were in poor general condition (ECOG ≥2) (Table 1). Among 81 patients in the validation cohort, 78 patients (96.3%) had diffuse large B‐cell lymphoma and the remaining three had peripheral T‐cell lymphoma (n = 2) and Burkitt's lymphoma (n = 1).Treatment characteristics are summarized in Figure S1. Of the 81 PCNSL patients in the validation cohort, 56 patients (69.1%) underwent chemotherapy as first‐line treatment, and MTX was the most commonly used drug (n = 51) as the frontline treatment, followed by high‐dose cytarabine (n = 36), rituximab (n = 3), and vincristine (n = 3). Radiotherapy was administered in 65 patients (80.2%). Radiotherapy was administered to the whole brain with a median dose (range) of 40 (13‐50) Gy, with or without a tumor‐bed boost with 11 (5‐18) Gy. The disease status at the end of follow‐up as well as causes of mortality in the validation cohort are shown in Figure S2.

**Figure 3:**
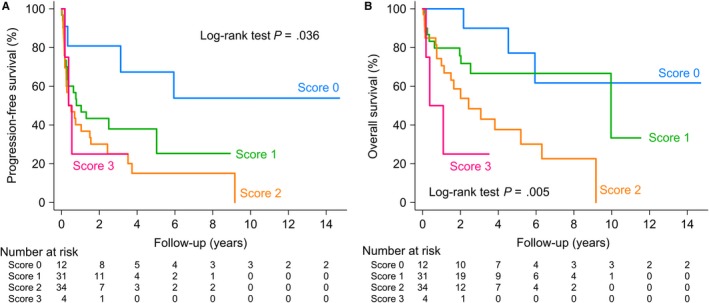
Kaplan—Meier estimates of (A) progression‐free survival and (B) overall survival in primary central nervous system lymphoma (PCNSL) patients with the Taipei Score (validation cohort)

Compared with the training cohort, the validation cohort had a higher proportion of ECOG ≥2 (53.5% vs. 71.6%; *P* = .012). The median PFS and OS for PCNSL patients were shorter than those of the training cohort, at 0.8 years (95% CI 0.4‐2.5) and 5.9 years (95% CI 2.5‐10.0), respectively. Figure 3 shows that our score can clearly distinguish those with better outcomes from those with poorer PFS (log‐rank test *P* = .036) and OS (log‐rank test *P* = .005) in the validation cohort. Furthermore, C‐statistic of the Taipei Score on the validation cohort was 0.60 (95% CI 0.51‐0.68) for PFS and 0.65 (95% CI 0.56‐0.74) for OS (Table S2).

### 3.6 IELSG, NB, MSKCC scores

The comparisons of prognostic models are presented in Table 4. We also tested the associations between IELSG, NB or MSKCC scores and PFS and OS in both the training cohort (Figure S3) and validation cohort (Figure S4). The IELSG and NB models showed poor separation for both PFS and OS. While there were significant differences in PFS in PCNSL patients by MSKCC scores in the training cohort (log‐rank test *P* = .003), higher scores did not associate with shorter PFS. Instead, patients in the class 2 appeared to have better PFS than patients in the class 1. Also, results of the C‐statistics showed that the IELSG, NB, and MSKCC score had insufficient discriminative ability, with the 95% confidence intervals of the C‐statistics crossed 0.5 for either PFS or OS in both cohorts. (Table S2).

**Table 4:** Comparison of prognostic models and distributions of risk groups

	Taipei Score	IELSG prognostic score	NB prediction score	MSKCC prognostic model
Risk factors				
Age	✓ (Age ≥80)	✓ (Age >60)	✓ (Age ≥60)	✓ (Age ≥50)
Performance status	✓ (ECOG ≥2)	✓ (ECOG ≥2)	✓ (ECOG ≥2)	✓ (KPS <70)
Deep brain involvement	✓	✓	—	—
Multifocal lesions or meningeal disease	—	—	✓	—
Elevated LDH serum level	—	✓	—	—
High CSF protein concentration	—	✓	—	—
Risk groups				
Low	0 risk factors	0‐1 risk factors	0 risk factors	Age <50
Intermediate	1 risk factors	2‐3 risk factors	1 risk factors	All others
High	2 risk factors	4‐5 risk factors	2 risk factors	Age ≥50 and KPS <70
Very high	3 risk factors		3 risk factors	
Distribution of risk groups				
In this database (training cohort) (n = 101)				
Low	16 (15.8%)	7/54 (13.0%)	9 (8.9%)	14 (13.9%)
Intermediate	41 (40.6%)	30/54 (55.6%)	35 (34.7%)	49 (48.5%)
High	37 (36.6%)	17/54 (31.5%)	42 (41.6%)	38 (37.6%)
Very high	7 (6.9%)		15 (14.9%)	
Published previously^9^, ^10^, ^11^				
Low		26/105 (24.8%)	8/77 (10.4%)	84/282 (29.8%)
Intermediate		56/105 (53.3%)	29/77 (37.7%)	125/282 (44.3%)
High		23/105 (21.9%)	28/77 (36.4%)	73/282 (25.9%)
Very high			12/77 (15.6%)	

Abbreviations: CSF, cerebrospinal fluid; ECOG, Eastern Cooperative Oncology Group performance score; IELSG, International Extranodal Lymphoma Study Group; KPS, Karnofsky performance status; LDH, lactic dehydrogenase; MSKCC, Memorial Sloan Kettering Cancer Center; NB, Nottingham‐Barcelona.

## 4 DISCUSSION

More than a decade after the development of the IELSG, NB and, MSKCC scoring systems, we have reevaluated their applicability to predict outcomes for patients in more recent periods. However, all the previous prediction models do not enable significant discrimination between the validation risk groups. In our cohorts, only three of the original five factors used in IELSG retained prognostic value for PFS (age, PS, deep brain involvement) and two factors for OS (ie, age and deep brain involvement). Based on this, a novel prognostic score, the Taipei Score, was established that identifies four different risk groups, depending on 0, 1, 2, or 3 risk factors. Furthermore, using an alternative age cutoff of 80 did appear to enhance the prognostic power of the Taipei Score since the application of original age cutoff (50 in MSKCC or 60 in IELSG) revealed a lack of separation between different risk‐stratified groups. Thus, our model outperformed the other three conventional models and provided risk stratification for both PFS (*P* < .001) and OS (*P* = .005).

Our cohort population is not identical to IELSG and MSKCC reports. We have summarized the distribution of risk groups in Table 4. Although the proportion of the intermediate‐risk group was similar, it appeared that there was a greater proportion of patients with high‐risk score and a smaller proportion of patients with low‐risk score in our cohort. In previous models, only 21.9% (IELSG cohort^9^) and 25.9% (MSKCC cohort^11^) of the patients were included in the high‐risk group, whereas the high‐risk group in our training cohort accounted for 31.5% (by IELSG model) and 37.6% (by MSKCC model) of patients. Interestingly, this did not negatively impact survival outcomes. Rather, the median OS reached 8.4 years, increasing remarkably when compared with previous reports.^9^, ^10^, ^11^ This substantiated the results reported by some specialized centers,^22^, ^23^, ^24^ as well as those from large national databases,^25^, ^26^, ^27^ indicating significant improvement in survival of PCNSL over past decades. The generalized improvement in patient outcomes may be attributed to several key reasons, such as the use of autologous stem cell transplant as consolidation therapy, availability of neutrophil growth factors and improved supportive care to bolster the administration of high‐dose MTX as well as multi‐agent chemotherapy, and prevalent use of recent‐generation imaging modalities, namely improved MRI and PET/CT scanners, allowing for accurate staging (eg, elimination of patients with non‐CNS involvement who may have previously been misdiagnosed with PCNSL). In our dataset, the discriminative ability of the three previous models cannot be reproduced, suggesting the need for validating their clinical relevance for PCNSL patients using more prospective studies in more recent periods.

Our newly developed model is dependent on three identified variables, including age, performance status, and deep brain involvement. After adjusting for confounding factors, patient age remained a significant prognostic factor for both OS and PFS. This is in accordance with the literature.^28^, ^29^ However, the traditional cutoffs, namely 50 and 60 years of age, failed to dichotomize survival outcome efficiently in our cohort, although the median age of our patients was similar to that reported in previous studies. Unlike prior prognostic models, the cutoff was 80 years in our data, much higher than that used in IELSG (60 years), NB (60 years) and MSKCC (50 years).^9^, ^10^, ^11^ This discrepancy may be explained by the evolving treatment and life expectancy of elderly PCNSL patients in more recent times, thereby blurring the line between patients separated using conventional cutoffs. Indeed, when grouping patients by time of diagnosis (before 1987 vs. 1987‐1997 vs. 1997‐2007 vs. after 2007), improving OS over time has been revealed for patients ≥60 years of age.^23^, ^24^, ^30^ Elderly PCNSL patients tend to benefit from the introduction of high‐dose chemotherapy.^30^, ^31^ However, patients aged 70 or older have been shown to be the exception of the generalized improvement in PCNSL survival over the last 40 years.^25^ We thus argue that old age defined by >50 or >60 should not be applied as exclusion criteria for treatment. We also recommend an alternative age cutoff in order to achieve better prognostication of PCNSL patients.

Our study also showed significance for PS and deep brain involvement as two other major prognostic factors. Poor PS has served as exclusion criteria in a wide variety of clinical trials^32^, ^33^, ^34^, ^35^ and has long been accepted as a powerful predictive factor among studies. In previous retrospective studies (IELSG, MSKCC, NB), PS consistently determined the survival of PCNSL patients.^9^, ^10^, ^11^ Contrary to our expectations, although PS (ECOG ≥2) is independently associated with PFS in our cohort, it was not a significant risk factor for OS in the multivariate analysis. This may be attributed to the strong interaction between PS and age, specifically that old age adversely affects organ function and reserve, as well as deep brain involvement— ECOG score is dependent on neurological status and hence influenced by deep brain involvement. The effect of PS on estimating OS was thus outweighed by the effect of age and deep brain involvement in the multivariate analysis. In terms of deep brain involvement, both earlier and recent findings concurred with ours that deep structure involvement is correlated with both OS and PFS independently.^9^, ^13^, ^31^

Except for age, PS and tumor location, we could not detect clinical significance of any prognostic factors proposed in previous models. In fact, some of these parameters (ie, CSF protein concentration and serum LDH level), though serving as significant risk factors in the IELSG model, are not readily available and even sometimes contraindicated in clinical practice. PCNSL patients often exhibit elevated intracranial pressure due to brain lesions accompanied by perifocal edema, which thereby prohibits routine lumbar puncture before the commencement of treatment. Indeed, there is a high percentage of missing values regarding this variable in the original IELSG study and many subsequent retrospective cohorts, leading to the failure of assigning a complete IELSG score.^9^, ^11^, ^12^, ^13^, ^14^ On the contrary, the Taipei Score is based on only three variables, namely, age, PS and tumor location, which are easily and regularly obtainable in clinical practice, therefore making it an amenable model for clinical use.

To evaluate the prognostic impact of other readily available parameters, we added hemoglobin,^19^, ^36^ C‐reactive protein,^19^, ^37^ and bilirubin levels^19^ into our analysis. Nevertheless, we did not detect significant correlation between these factors and clinical outcomes in this study. Further validation of the prognostic value of these factors by prospective and large studies may be needed before their common use in clinical practice.

The discriminative capacity of our prognostic model was reproduced in the independent validating cohort. Similarly, our simplified Taipei Score model outperformed all the three conventional models (IELSG, NB, and MSKCC) in the validating set. Notably, though we used the same inclusion and exclusion criteria, the distribution of baseline characteristics was very different between the training and validation cohort. Compared with the training cohort, there was relatively a lower rate of very elderly patients (≥80) and a higher rate of patients with poor PS (ECOG ≥2) in the validating cohort. However, such discrepancy in the predicting variables between the two groups is thought to reflect the true picture of very wide demographic and geographic variations in routine clinical practice.

The major limitation of this study is its retrospective nature, which may introduce inherent selection bias by recruiting PCNSL patients who were diagnosed and followed at tertiary medical centers only. Furthermore, our patient cohorts were mainly Asian, which may partly explain the failure of the three models developed in Western populations, to associate with PFS and OS in our cohorts. Nevertheless, to the best of our knowledge, our study provides the largest evidence for the validation of IELSG, NB and MSKCC scores in Asian patients. Moreover, in this study cohort, there was a substantial proportion of patients with incomplete cytogenetic and molecular data. Therefore we were unable to further identify patients with double‐hit or triple‐hit lymphomas as well as to subclassify CNS diffuse large B‐cell lymphoma according to Hans criteria.^38^ However, although molecular and cytogenetic studies might provide additional prognostic information, the process could be expensive and laborious, which potentially limits the accessibility and utility of a prognostic model. On the contrary, Taipei Score was based only on three clinical parameters, and was therefore simple and intuitive to be applied in clinical practice. Finally, in a retrospective study, the choice of combination chemotherapy regimen was at the physician's discretion and was potentially affected by patients' will rather than standard care for PCNSL. The heterogeneous treatments received by the study populations may affect the evaluation of potential prognostic factors, resulting in null findings. These heterogeneous circumstances, however, reflect the current clinical scenarios of treating PCNSL patients because there has long been controversy regarding optimal regimens for PCNSL.^4^, ^39^

In summary, the Taipei Score is a simple model that discriminates PFS and OS for PCNSL patients. The score may offer disease risk stratification and help facilitate clinical decision‐making.

## CONFLICT OF INTEREST

The authors declare that they have no conflict of interest.

## AUTHORS' CONTRIBUTIONS

C‐JL and C‐HL had full access to all of the data in the study and take responsibility for its integrity as well as the accuracy of the data analysis. C‐HL and C‐JL designed the study. C‐FY, C‐MY, C‐HL, S‐YL, and C‐JL acquired the data and performed statistical analysis. C‐FY, H‐YW, S‐YL, C‐KT, ASK, and P‐SK provided the final interpretation of the results. C‐JL and C‐HL drafted the manuscript. C‐KT, Y‐CL, C‐FY, J‐PG, Y‐CH, and L‐TH made critical revisions to the manuscript for important intellectual content. C‐MY, P‐MC, and J‐HL provided administrative, technical, and material support. C‐HL was the study supervisor. C‐HL acts as a guarantor and accepts responsibility for the integrity of the work as a whole. All authors have read and approved the final manuscript.

## Data Availability

The datasets used and analyzed in this study are available from the corresponding author on reasonable request.
