# Supplementary figures and images for: A new prognostic score for disease progression and mortality in patients with newly diagnosed primary CNS lymphoma
Source: Cancer Med. 2020 Feb 3;9(6):2134–45. doi: 10.1002/cam4.2872 (PMC7064125; doi:10.1002/cam4.2872)

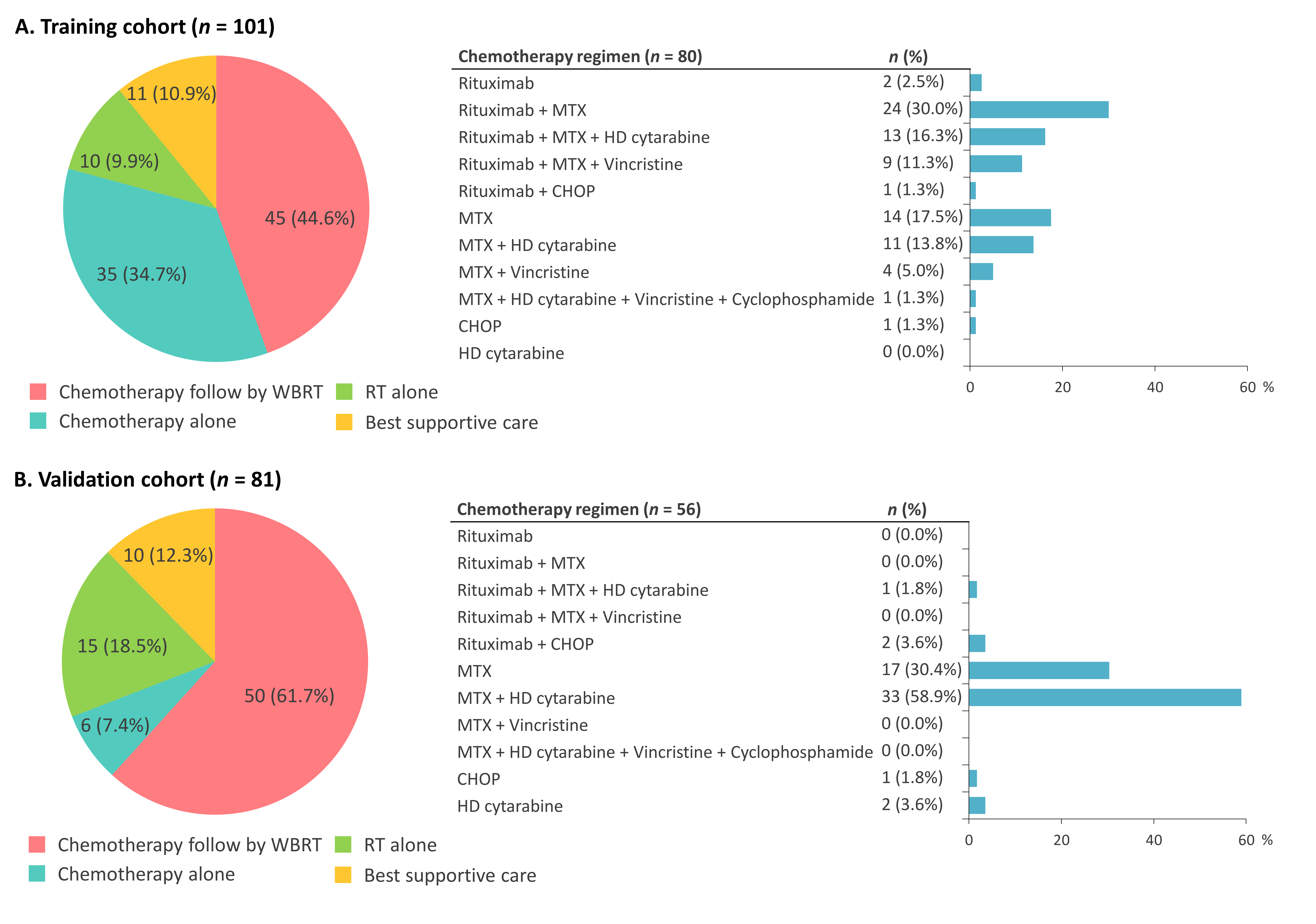

Supplement: Supplementary file 1 [file CAM4-9-2134-s001.tif]

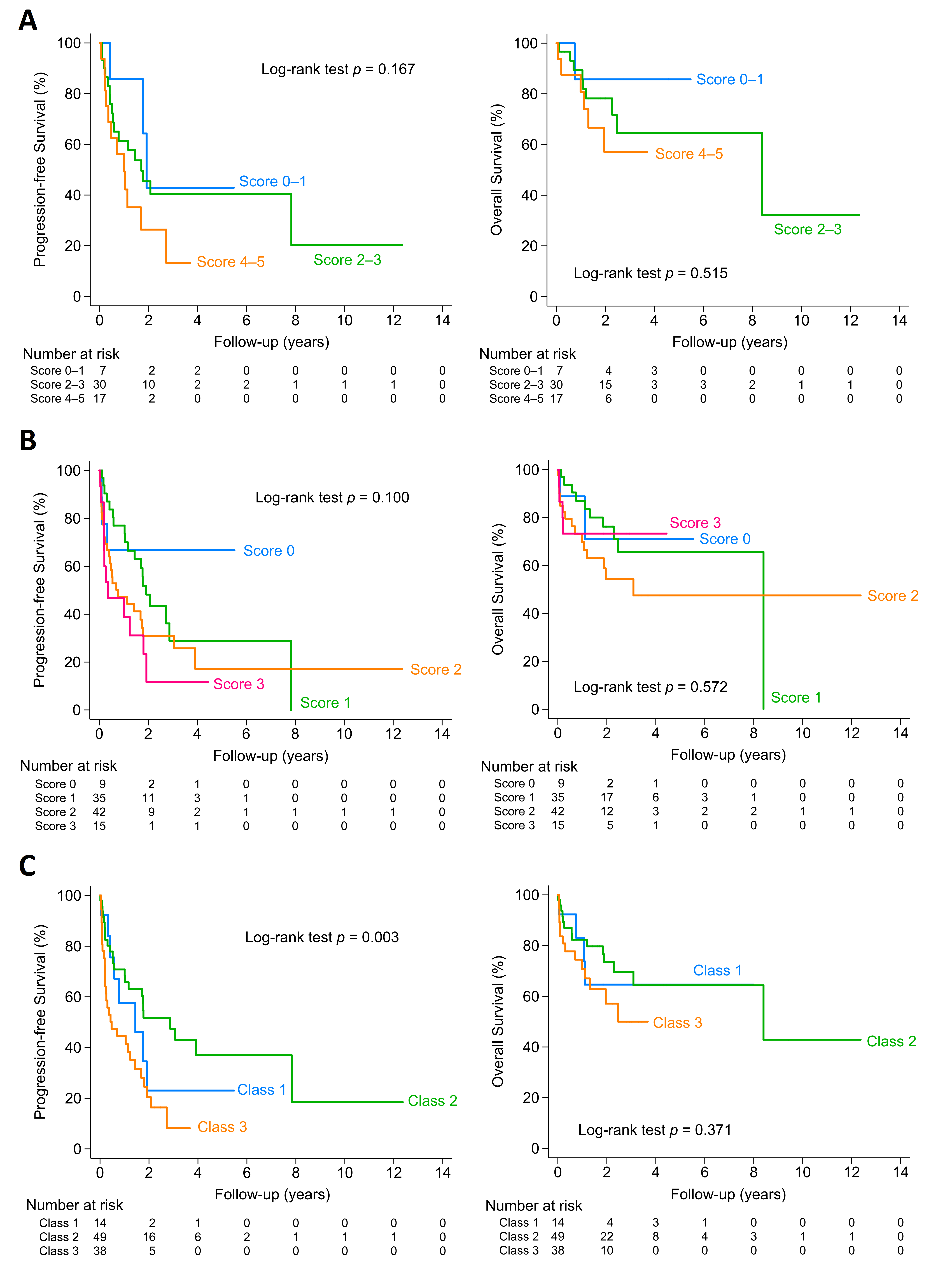

Supplement: Supplementary file 3 [file CAM4-9-2134-s003.tif]

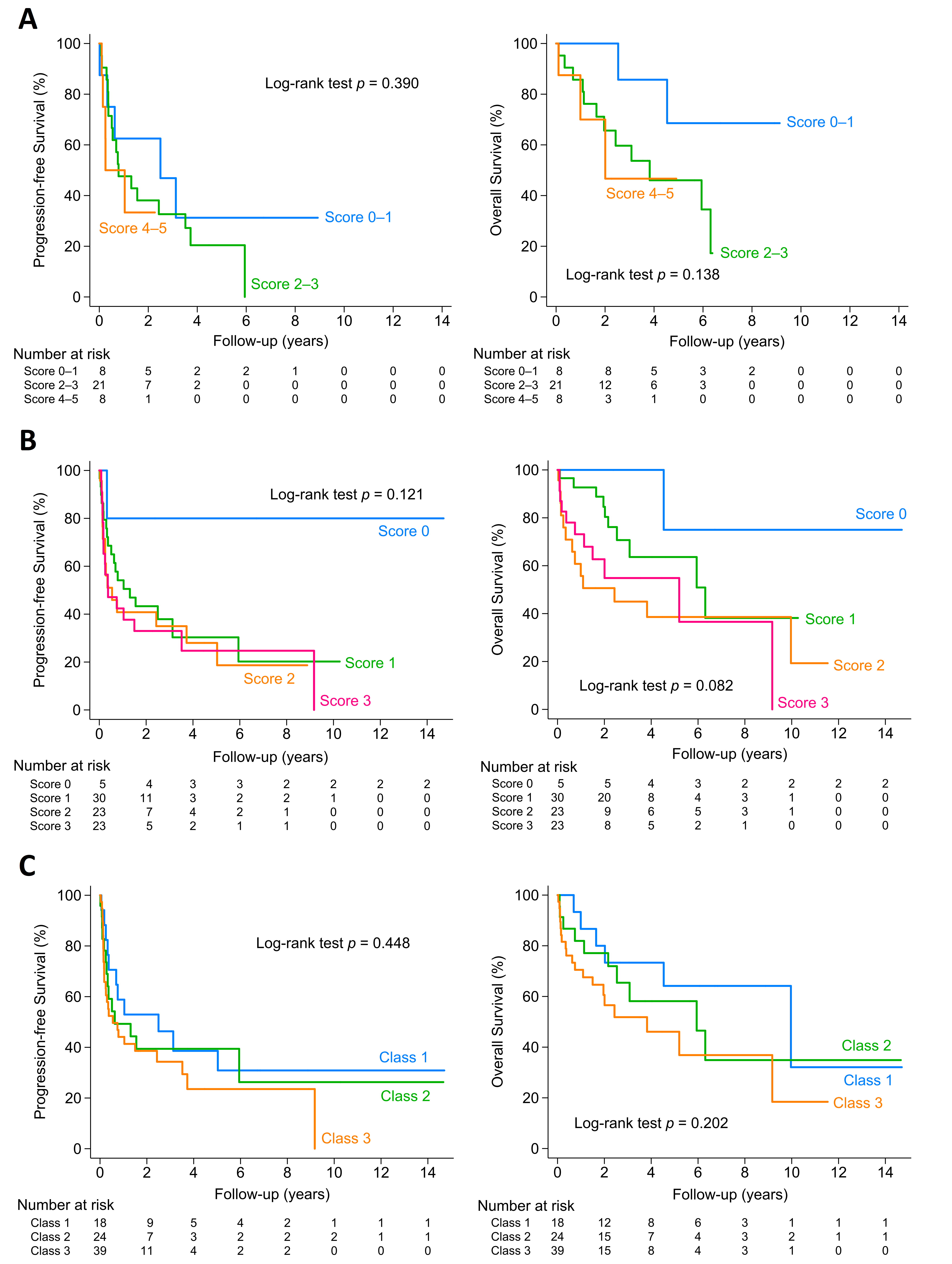

Supplement: Supplementary file 4 [file CAM4-9-2134-s004.tif]
