# Supplementary material for: A new prognostic score for disease progression and mortality in patients with newly diagnosed primary CNS lymphoma
Source: Cancer Med. 2020 Feb 3;9(6):2134–45. doi: 10.1002/cam4.2872 (PMC7064125; doi:10.1002/cam4.2872)
Supplement: Supplementary file 5 [file CAM4-9-2134-s005.docx]

**Supplemental Table 1. Baseline characteristics of PCNSL patients**

| **Characteristics** | **Training cohort**  ***n* = 101** | **Validation cohort**  ***n* = 81** | ***P* value** |
| --- | --- | --- | --- |
|  | ***n* (%)** | ***n* (%)** |  |
| **Taipei Score** |  |  |  |
| 0 | 16 (15.8) | 12 (14.8) | 0.869 |
| 1 | 41 (40.6) | 31 (38.3) |  |
| 2 | 37 (36.6) | 34 (42.0) |  |
| 3 | 7 (6.9) | 4 (4.9) |  |
| **IELSG prognostic score** |  |  |  |
| 0–1 | 7/54 (13.0) | 8/37 (21.6) | 0.410 |
| 2–3 | 30/54 (55.6) | 21/37 (56.8) |  |
| 4–5 | 17/54 (31.5) | 8/37 (21.6) |  |
| **NB prediction score** |  |  |  |
| 0 | 9 (8.9) | 5 (6.2) | 0.084 |
| 1 | 35 (34.7) | 30 (37.0) |  |
| 2 | 42 (41.6) | 23 (28.4) |  |
| 3 | 15 (14.9) | 23 (28.4) |  |
| **MSKCC prognostic model** |  |  |  |
| Class 1 | 14 (13.9) | 18 (22.2) | 0.031 |
| Class 2 | 49 (48.5) | 24 (29.6) |  |
| Class 3 | 38 (37.6) | 39 (48.2) |  |

IELSG, International Extranodal Lymphoma Study Group; NB, Nottingham-Barcelona; MSKCC, Memorial Sloan Kettering Cancer Center
