# Supplementary material for: A new prognostic score for disease progression and mortality in patients with newly diagnosed primary CNS lymphoma
Source: Cancer Med. 2020 Feb 3;9(6):2134–45. doi: 10.1002/cam4.2872 (PMC7064125; doi:10.1002/cam4.2872)
Supplement: Supplementary file 6 [file CAM4-9-2134-s006.docx]

**Supplemental Table 2. Harrell’s C statistics of the four evaluated models of PCNSL**

|  | **PFS** |  | **OS** |
| --- | --- | --- | --- |
|  | **C-statistic (95% CI)** |  | **C-statistic (95% CI)** |
| **Training cohort** |  |  |  |
| Taipei Score | 0.67 (0.61–0.74) |  | 0.67 (0.59–0.76) |
| IELSG | 0.59 (0.50–0.68) |  | 0.58 (0.44–0.72) |
| NB | 0.60 (0.53–0.68) |  | 0.59 (0.49–0.68) |
| MSKCC | 0.61 (0.54–0.68) |  | 0.57 (0.47–0.67) |
| **Validation cohort** |  |  |  |
| Taipei Score | 0.60 (0.51–0.68) |  | 0.65 (0.56–0.74) |
| IELSG | 0.57 (0.44–0.70) |  | 0.64 (0.52–0.76) |
| NB | 0.57 (0.49–0.66) |  | 0.66 (0.57–0.75) |
| MSKCC | 0.55 (0.48–0.63) |  | 0.60 (0.51–0.69) |

IELSG, International Extranodal Lymphoma Study Group; NB, Nottingham-Barcelona; MSKCC, Memorial Sloan Kettering Cancer Center; CI, conﬁdence interval; PFS, progression-free survival; OS, overall survival.
